# Portable wireless electrocorticography system with a flexible microelectrodes array for epilepsy treatment

**DOI:** 10.1038/s41598-017-07823-3

**Published:** 2017-08-10

**Authors:** Kejun Xie, Shaomin Zhang, Shurong Dong, Shijian Li, Chaonan Yu, Kedi Xu, Wanke Chen, Wei guo, Jikui Luo, Zhaohui Wu

**Affiliations:** 1 Key Laboratory of Micro-nano Electronic Devices and Smart Systems of Zhejiang Province, College of Information Science & Electronic Engineering, Zhejiang University, Hangzhou, 310027 China; 2 College of Computer Science, Zhejiang University, Hangzhou, 310027 China; 3 Key Laboratory of Biomedical Engineering of Education Ministry, Department of Biomedical Engineering, Zhejiang University, Hangzhou, 310027 China; 4 Qiushi Academy for Advanced Studies (QAAS), Zhejiang University, Hangzhou, 310027 China; 5 College of Electron Infor., Hangzhou Dianzhi University, 2nd Street, Hangzhou, 310018 China; 6 Institute of Renewable Energy & Environmental Technology, University of Bolton, Deane Road, Bolton, BL3 5AB United Kingdom

## Abstract

In this paper, we present a portable wireless electrocorticography (ECoG) system. It uses a high resolution 32-channel flexible ECoG electrodes array to collect electrical signals of brain activities and to stimulate the lesions. Electronic circuits are designed for signal acquisition, processing and transmission using Bluetooth Low Energy 4 (LTE4) for wireless communication with cell phone. *In-vivo* experiments on a rat show that the flexible ECoG system can accurately record electrical signals of brain activities and transmit them to cell phone with a maximal sampling rate of 30 ksampling/s per channel. It demonstrates that the epilepsy lesions can be detected, located and treated through the ECoG system. The wireless ECoG system has low energy consumption and high brain spatial resolution, thus has great prospects for future application.

## Introduction

Brain-computer interfaces (BCI) for medical and non-medical applications has made substantial progress in last few decades, and has been applied in various areas, such as control of motion and electrical stimulation treatment of paralyzed persons etc.^1^. For medical and clinic applications, such as identification of epileptic foci for clinical purpose, devices with microelectrodes to record electrocorticogram (ECoG) signals are necessary. An array of microelectrodes placed directly on the cortical surface of a brain could record spatial distribution of neural activity with high accuracy^2^, and provide detailed information of cognitive behavior of a brain. Therefore an array of microelectrodes could serve not only as basis for weakly-invasive high-performance BCIs, but also as ECoG electrodes which have broad medical applications. For instance, it could be used to stimulate the areas of a brain to assess the risk of eloquent cortex damage during a surgical resection, as the control auxiliary devices to help patients to move their injured limbs, as a meditation for drug addicted person etc. The ECoG electrodes array are particularly useful for those with motor disabilities who need a communication device to guide the mobility, and those paralyzed who use ECoG to control their muscle etc.^3^. For epilepsy patients, it is important to know when they are under attack. Therefore, a portable wireless ECoG system is convenient for doctors to monitor patients’ conditions.

ECoG microelectrodes normally are a flexible planar structure. The electrodes are small in dimension, providing much higher spatial resolution, better signal-to-noise ratio (SNR) and broader bandwidth compared to electroencephalograms (EEG) systems, and they have more stable measurements compared to micro-needle electrode arrays^4^. ECoG recording system has been regarded as a promising tool for BCIs-based medical and welfare applications^5^. However, most of the ECoG system requires wire connections for communication, which is not portable. A wireless ECoG system is therefore desirable and necessary. For long-term, real-time and portable observation and medical intervention of disordered patients, a mobile phone based ECoG system with wireless communication and control capability would be practically useful. This paper reports a wireless ECoG recording system with a flexible and biocompatible electrode device with an array of 32-microelectrodes. Based on a rat’s epilepsy treatment experiment, we show the ECoG system can record brain activity and perform electric stimulation wirelessly, demonstrating its potential for broad applications.

## Results

### Flexible wireless ECoG system

**Figure 1:**
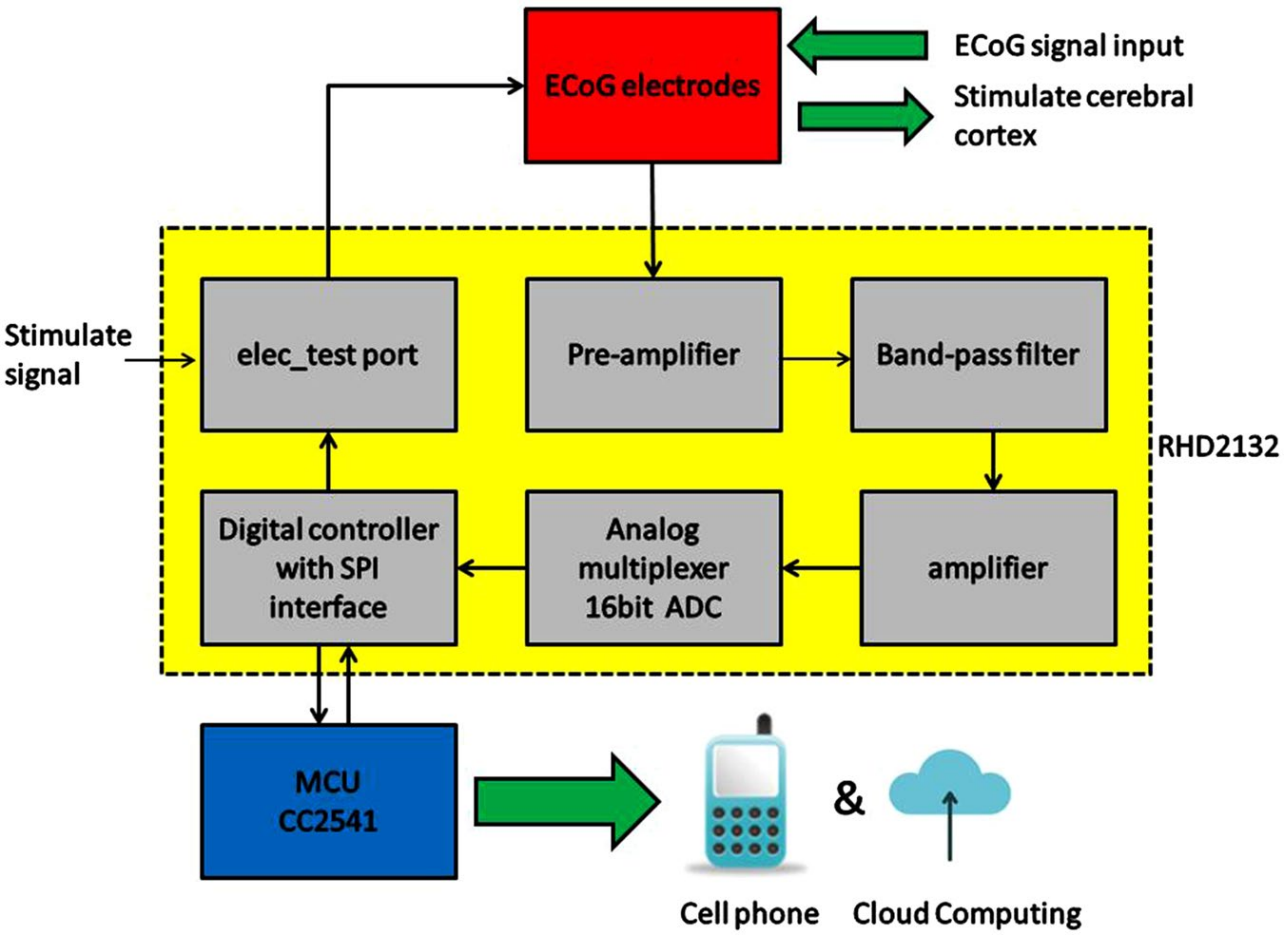
Diagram of the ECoG system. The red block is the ECoG electrode device with an array of 32 flexible microelectrodes, which are used to record ECoG signals of a brain or apply stimulation electrical signal to suppress epilepsy; the yellow block is the electronic circuits which acquire and process ECoG signals; the blue block is the microcontroller unit controlling the ECoG system, and communicate with cell phone ad cloud system for data processing etc.

Figure 1 shows block diagram of the wireless ECoG recording system developed. The ECoG signals are collected through an array of 32 flexible microelectrodes. After amplifying and filtering process, signals are converted into digital ones by a 16-bit ADC (analog to digital converter, RHD2132), and sent to the microcontroller unit (MCU) CC2541 through a signal processing interface (SPI). The signals are then transmitted to a cell phone by Bluetooth Low Energy (BLE). The cell phone and Cloud system will analyze the ECoG signals and make a decision if it is necessary for epilepsy treatment. Stimulation electrical signals are applied to epileptic cortex lesions by microelectrodes to suppress epilepsy if required.

The Bluetooth Low Energy was used for wireless communication between the ECoG device and cell phone. A TI CC2541 BLE chip was used as the microcontroller unit (MCU) to control the function of RHD2132 (Digital Electrophysiology interface chips). Barron circuit was used to convert differential signals to single-ended signals, and for impedance matching. The amplitude of ECoG signals is typically at μV level with the frequency under 100 Hz, which usually is the bandwidth of useful brain signal^6, 7^, therefore the recording system requires an amplifying and filtering circuit to improve SNR. A RHD2132 was applied as the pre-preamplifier. As the voltage range is 3.2–3.6 V for RHD2132, and 2.0–3.6 V for CC2541, a LDO (low dropout regulator) circuit and TPS79933 were chosen to regulate and stabilize the voltage at 3.3 V for both the circuits. CC2541’s BLE RF transceiver may receive a lot of high frequency noises and pull down the supply voltage, whereas RHD2132 needs a stable voltage source and clear inputs. To solve this, two magnetic beads were used to separate the power and ground between RHD2132 and CC2541 to avoid their interaction effectively. The details of the electronic circuits can be found from Figures S1–S3 in Supplement.

**Figure 2:**
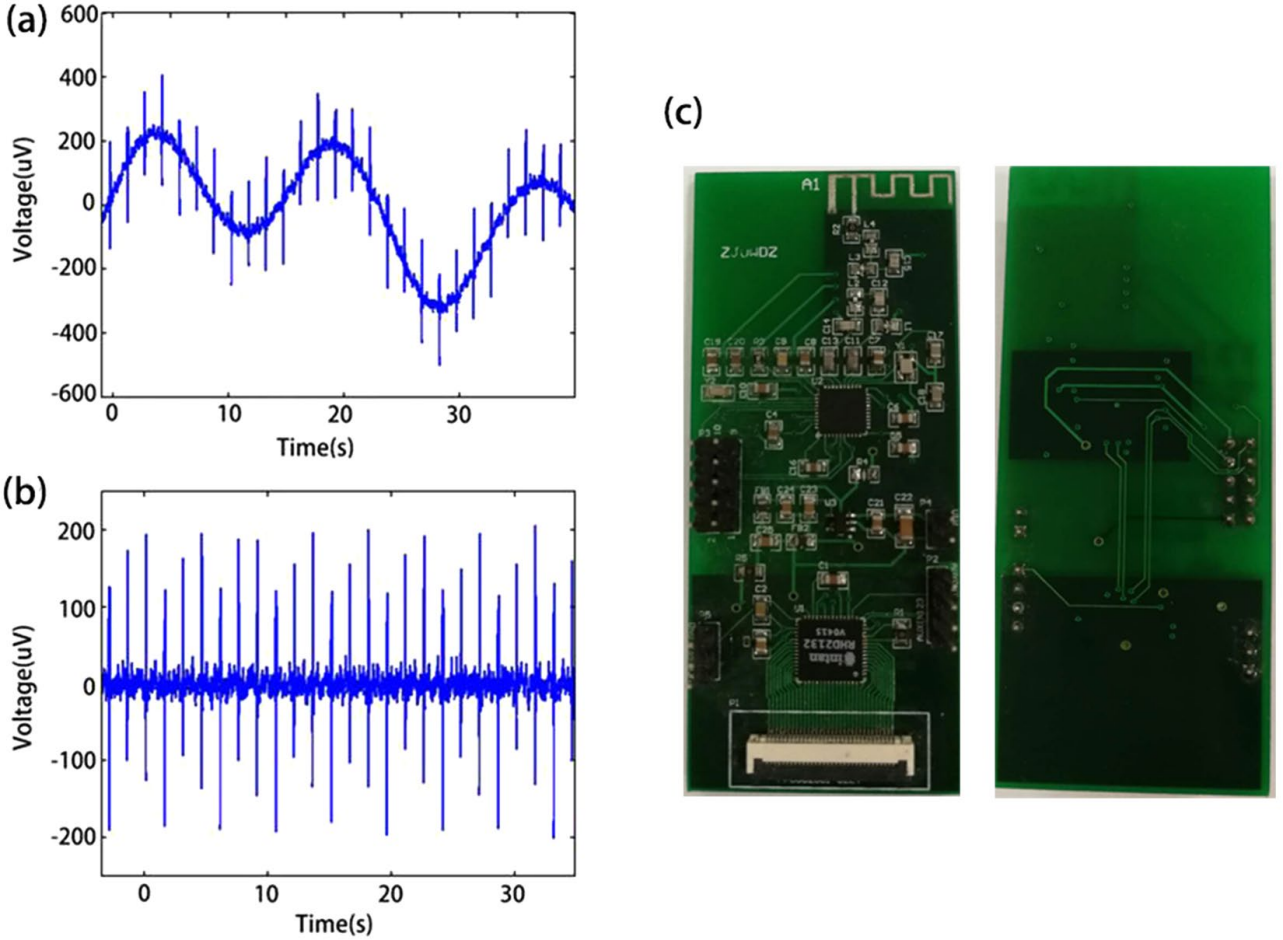
Electrical signals and electronic circuits. (**a**) The electrical signal from a brain wave generator before filtering, it contains a low frequency noise; (**b**) the signal waveform after filtering which shows the removed of low frequency noise, and (**c**) PCB photos of both top and bottom layers.

A band-pass filter was designed to filter high frequency and low frequency interferences to improve SNR that is embedded in RHD2132. The frequency of useful ECoG signals are usually between 0.1 to 7500 Hz, therefore the bandwidth of the band-pass filter was set between 0.1 to 7500 Hz. To verify the reliability of the circuit, we verified the circuits with signals generated from a brain wave generator(a 128-Channel Neural Signal Simulator produced by Cyberkinetics Inc.) which includes spike signals and low frequency noise as shown in Fig. 2a. Figure 2b is the waveforms after filtering, clearly showing that the low frequency interference has been filtered completely, and indicating the effectiveness of the circuits.

All the circuits were arranged into four layers on a PCB for miniaturization with better SNR and functions as shown in Fig. 2c. The top and bottom layers are to receive and process signals. Between them, there are a power electronics layer and a ground layer with a large area of copper film to suppress noises from the power and ground layers due to the change of signals. A distributed capacitance between power plane and ground plane was designed to restrain the coupling of spike signals and to decrease the noises from the power line. As radio frequency circuits are sensitive to Electro Magnetic Interference (EMI), all wires have been designed as short as possible to reduce the wire inductance and associated noises brought in. Furthermore, polyimide(PI) printed circuit is used to realize this flexible wireless ECoG system as shown in Figure S4 in Supplement. The CC2541 and RHD 2132 are soldered on both side of the PI board to save space.

As the computing power of cell phones is limited, we developed a cloud model to process and recognize real meaning of ECoG signals from a brain. Massive distinct ECoG data can be uploaded to cloud system by APP for processing. The cloud system can easily extract potential patterns and hidden information in ECoG signals of a patient with epilepsy and improve the classification algorithm iteratively by analyzing and integrating large amounts of data. The APP can also receive, store data locally, and display it through different channels in real-time or within a specific time frame. The APP in a cellphone can invoke the pre-trained epilepsy detecting model to identify whether the patient is suffering from epilepsy or not. If it detects signals of epilepsy, it can send a treatment instruction to the user.

### Flexible microelectrodes array

**Figure 3:**
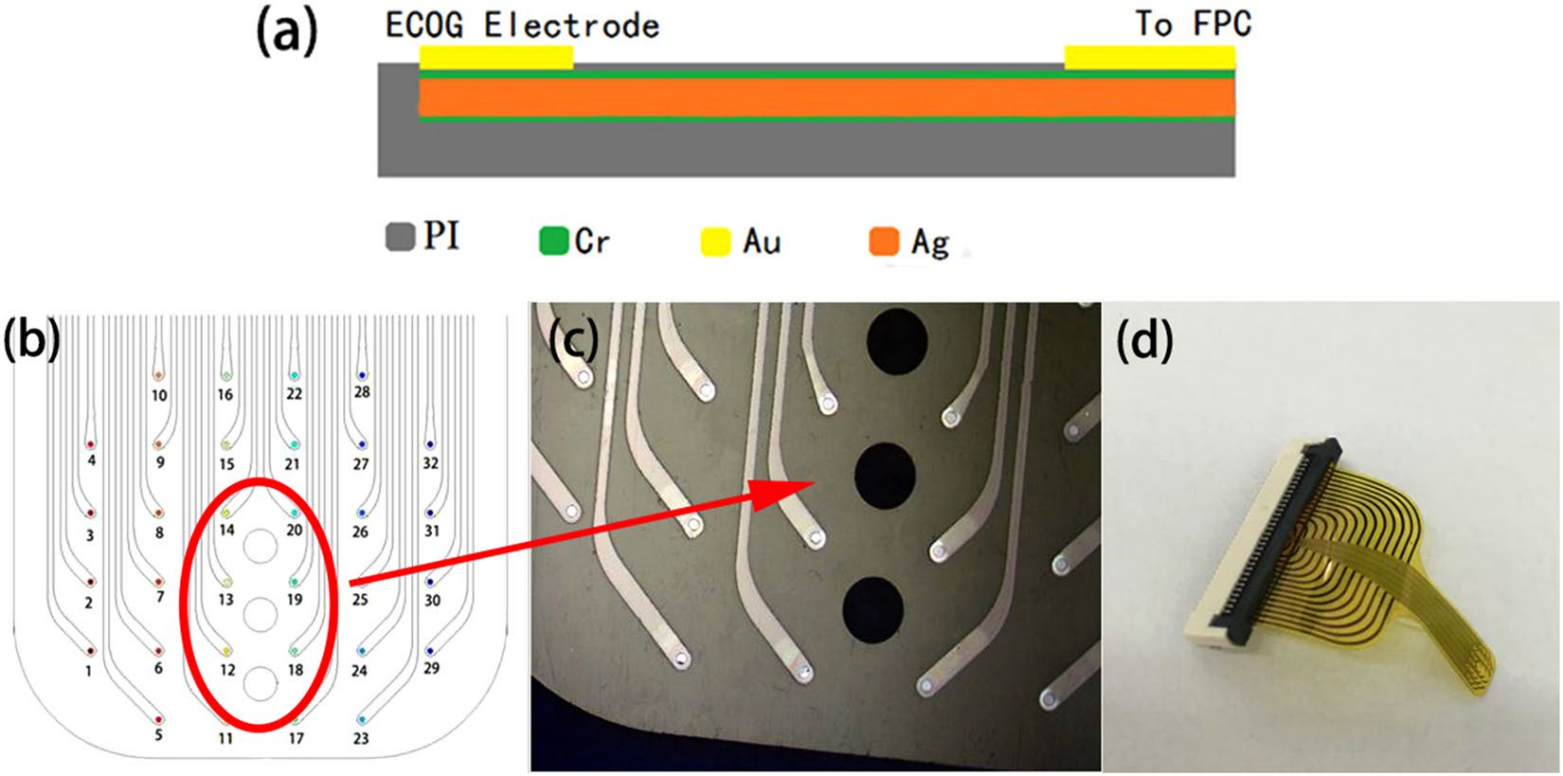
Flexible microelectrodes array. (**a**) Cross sectional view of a microelectrode, (**b**) ECoG electrode device with 32 microelectrodes, (**c**) a microscope image of the microelectrodes and three large holes for drug injection, and (**d**) a photo of the ECoG electrode device, clearly showing its flexibility.

The ECoG device has an array of flexible microelectrodes, and could fit to wrinkled surface of the cortex conformally^8, 9^, thus it has better contact with more neurons^10–12^ and has better signal to noise ratio compared to traditional ECoG electrode devices with rigid substrates. Figure 3 is a schematic and photo of the microelectrodes, and the flexible and pluggable ECoG device developed for neural signal recording and electrical stimulation. The electrode device has an array of 32 microelectrodes, with a total width of 16.3 mm and a length of 24.8 mm. The width of a microelectrode is 100 μm at the tip, and each microelectrode has an open surface of 50 μm in diameter at the tip for electrical contact. The 32-microelectrodes array could cover most area of a rat brain, including the important subdomains. There are three large holes of 300 μm in diameter (three black dots in the photo of Fig. 3c) in the ECoG electrode device that are for drug injection.

As the microelectrodes of ECoG device are for implant in brain, their biocompatibility, flexibility and reusability etc have been considered in design and fabrication. Figure 3a shows a cross sectional view of a microelectrode of the ECoG device. It is made on a polyimide (PI) film of 60 μm with excellent flexibility and biocompatibility^9, 13^. The metal layer consists of Cr/Ag/Cr three layers with excellent flexibility and good conductivity. A contact window is open for electrical contact for each microelectrode. The microelectrodes were connected with a flexible printed circuit (FPC) board connector to form the ECoG electrode device as shown in Fig. 3(d). As it can be seen that the ECoG electrode array is flexible, and can fit well with wrinkled cortex of a brain, which ensures to record distinct neural signals with high SNR.

The impedance of each microelectrode was assessed at 1 kHz in saline with DF-I (IMP-2, Bak Electronics Inc, CN) to see their suitability for the implant. The impedance of all the 32 microelectrodes is below 120 kΩ with an average value of ~22.7 kΩ, much smaller than 600 kΩ required by implantation^14^, thus it can be used for *in-vivo* experiments. To test its stability and anti-corrosion properties in biofluid, the flexible microelectrodes were immersed in a 5% saline for 5 days. Close inspection and electrical measurement showed that there is no corrosion and impedance change at all.

### ECoG system *in-vivo* testing

**Figure 4:**
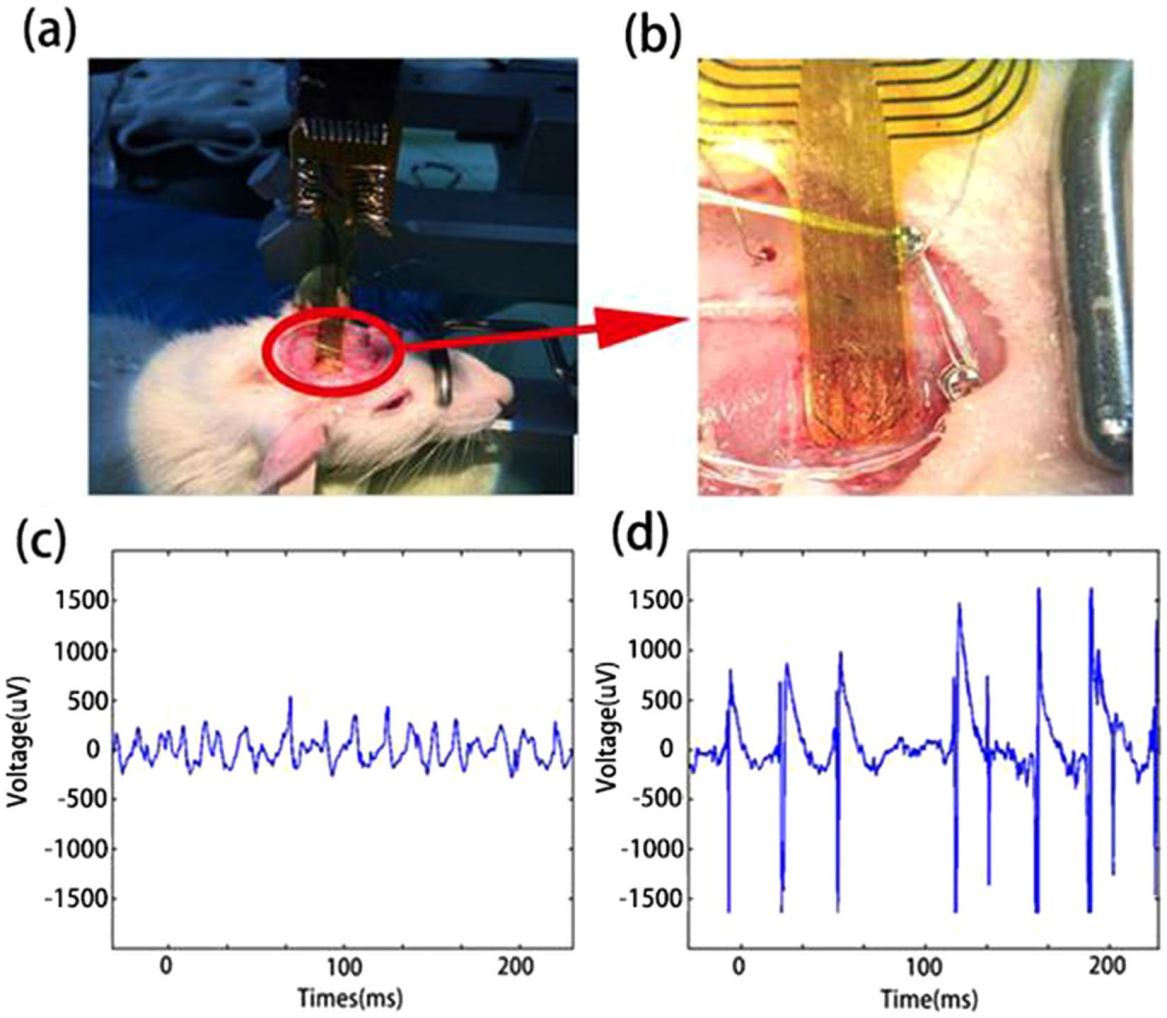
Implanted flexible ECoG electrode device and ECoG signals. A photo of the flexible electrode array placed on the left hemisphere of the brain of a Sprague-Dawley rat (**a**), zoomed-in photo of the microelectrodes (**b**), details of the zoomed-in waveforms for a rat at normal (**c**) and epilepsy (**d**) conditions.

The developed ECoG system was assessed with animal experiments. Craniotomy was conducted on a healthy adult male Sprague-Dawley rat to expose its cortex and the sterile microelectrode array was placed to cover the left primary sensory cortex of the rat brain as shown in Fig. 4a and b. The normal rat brain signal is shown in Fig. 4c with a typical signal amplitude less than 50 μV. The rat was then injected with penicillin (7.6 × 106 μ/kg) to induce epilepsy^15^. The recorded corresponding ECoG signal is shown in Fig. 4d, exhibiting obvious epilepsy spikes with large signal amplitude over 150 μV. The results clearly demonstrated the ability of the ECoG microelectrodes to record brain electrical signals. The rat ECoG signals from the 32 microelectrodes are shown in Figure S5 in Supplement.

**Figure 5:**
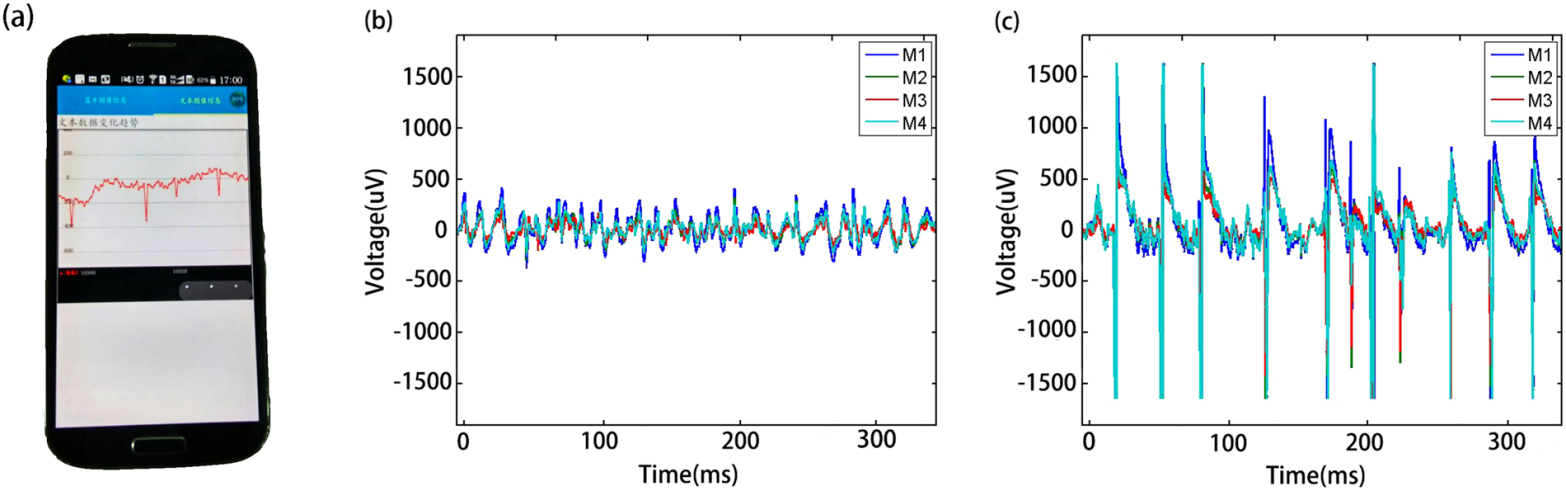
ECoG data show (**a**) in Cell phone, (**b**) and (**c**) in cloud with normal and epilepsy rat.

A cellphone was used to receive ECoG signals and display the information through APP, wireless communication and cloud system, with one channel result shown in Fig. 5a. Due to the limited graphics function of the cellphone, only some characteristic points of the signal are displayed. Figure 5b and c show the processed ECoG signals of the rat at normal and epilepsy conditions from four microelectrodes which contact the epilepsy lesions. Compared with the normal state (Fig. 5b), the ECoG signal of the rat under epilepsy (Fig. 5c) has spike signal with very large amplitude, typical characteristic of epilepsy. We could use data mining and pattern recognition algorithm to extract potential information through the cloud, however there are many patterns which is difficult to clearly recognized at the moment.

As the computing power of cellphone is limited, we developed a cloud model to process and recognize the real meaning of brain signals shown in Figure S6 in Supplement, and then realize the brain-cellphone interaction, which is under development^16^. The APP in cellphone can invoke the pre-trained epilepsy detecting model to identify whether the patient is suffering from epilepsy. Besides, massive distinct ECoG data can be uploaded to cloud system by APP for processing. The cloud system can easily extract potential patterns in ECoG signals of a patient with epilepsy and improve the classification algorithm iteratively by analyzing and integrating large amounts of data. The APP can also receive, store data locally, display it through different channels in real time or within a specific time frame. If it detects a wave indicating the epilepsy, it can send a treatment instruction to the user.

### ECoG system for rat epilepsy location and stimulation

**Figure 6:**
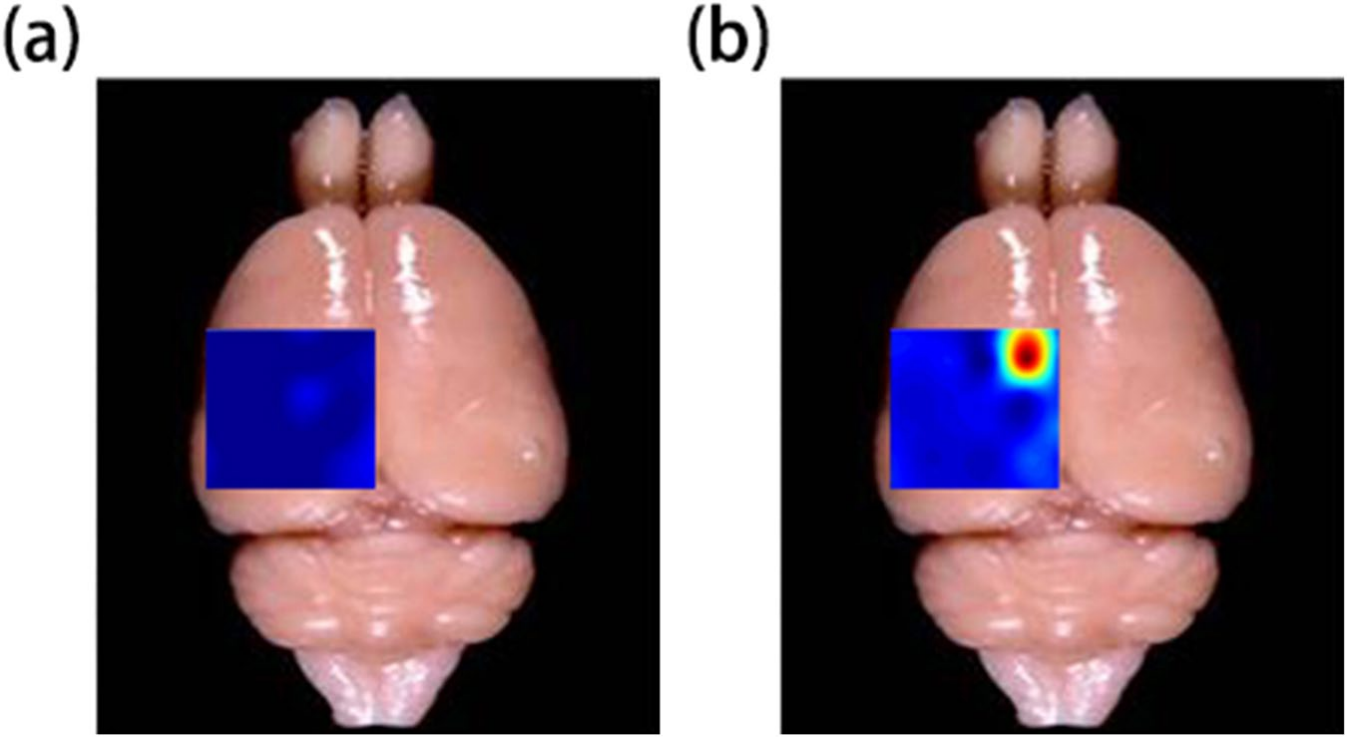
Mapping of ECoG signal amplitudes on the rat brain. Mappings of ECoG signal amplitudes from a rat brain under normal state (**a**) and epilepsy state (**b**). The red area has the highest amplitude, i.e. most active under epilepsy, which allows us to identify the epilepsy lesions in the brain.

Based on the recording results and signal processing, we can map out the amplitude of ECoG signals over the measured areas of the rat’s brain, and locate the exact epilepsy lesions. Figure 6a and b show the mappings of the brain electrical signal amplitudes measured under normal state and epilepsy state. The red area with the highest amplitude is the most active area under epilepsy and could be identified to be the epilepsy lesions.

**Figure 7:**
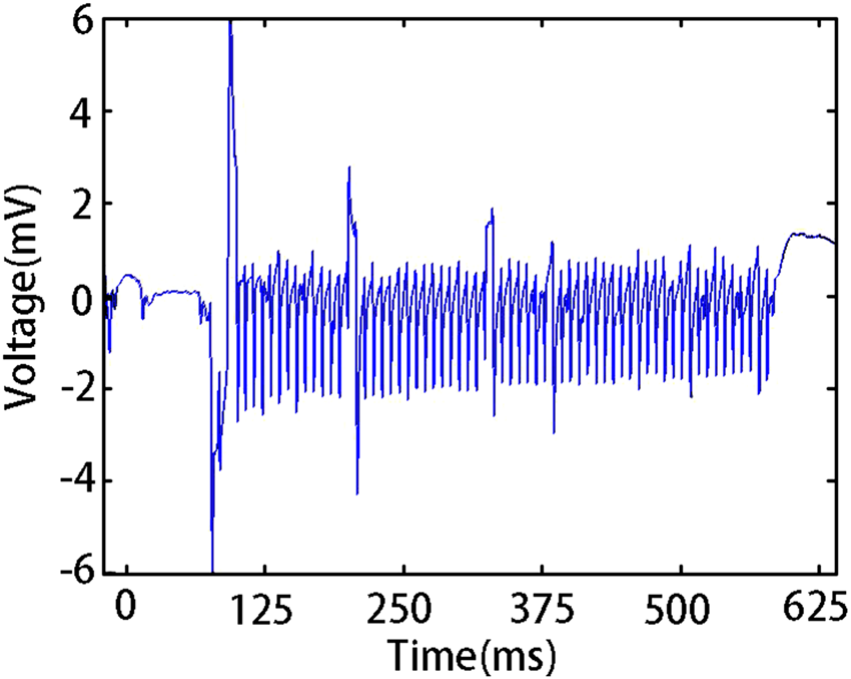
Response of the rat under electrical stimulation. Response ECoG signal of a rate when electrical stimulation is applied, which achieves an epilepsy condition, demonstrating the ability of the ECoG device for recording of brain wave signals and for providing electrical stimulation treatment.

Nevertheless, it also could stimulate at the epilepsy nidus after location. Indeed, we conducted this experiment to see if it is viable to achieve epilepsy using constant current electrical stimulation which is widely adoped^17^. Figure 7 shows the recorded response of the rat brain when 100 uA constant current electrical stimulation was applied, and the ECoG signal amplitude of epilepsy spikes reaches mV level. The results clearly demonstrated that the developed ECoG system could apply stimulation at epilepsy nidus. Maybe in the future we could develop a stimulation method how to suppress epilepsy.

## Discussion

Table 1 is the comparison of our ECoG system with other wireless systems. Our system has a higher sampling rate, which is important to obtain detailed and accurate information of the waveform of brain signals. Our system has a larger bandwidth which allows collecting more signals and information. Other advantages include high transmission rate and low power consumption that ensure better communication for a long time.

**Table 1:** Comparison of our system and other BCI systems.

	Lin^18^	Hirita^19^	Corinne^20^	Arezu^21^	Elena^22^	This work
Type	EEG	ECoG	ECoG	EEG	ECoG	ECoG flexible
Sampling Frequency	512 Hz	1 kHz	1 kHz	1 kHz	25 kHz	30 kHz
Number of channels	1	128	64	256	124	32
Bandwidth	0.1–100 Hz	0.1–1000 Hz	0.5–300 Hz	1–5000 Hz	1–150 Hz	0.1–7500 Hz
Resolution	12 bit	12 bit	12 bit	N/A	N/A	16 bit
Input referred noise	N/A	2.8 μV_rms_	1 μV_rms_	7.99 μV_rms_	N/A	2.4 μV_rms_
Power consumption	3.7 V Li Battery	300 mW	75 mW	12.9 μW	N/A	44.1 mW
Wireless Data rate	3 Mbps	400 kbps	450 kbps	N/A	N/A	2 Mbps
Wireless type	Bluetooth 2.0 + EDR	2.45 GHz	MICS band 402–405 MHz	ZigBee	N/A	BLE (Bluetooth Low Energy)
Device size	4 × 2.5 × 0.6 cm^3^ & 6.4 × 4.4 × 1 cm^3^	2 × 3 × 0.25 cm^3^ & 6 × 6 × 0.8 cm^3^	5 cm diameter Antenna: 10 cm^2^	30 × 22 × 15 mm^3^	150 mm^2^	1.8 × 2.4 × 1.1 cm^3^

Since electroencephalogram can directly map human actions, consciousness and emotions, after data pre-processing, feature extraction and pattern recognition, ECoG data can be used in clinical medicine for the treatment of anxiety, insomnia, Alzheimer’s disease, brain tumors, epilepsy and other diseases^23^. Furthermore, large amounts of ECoG data are very useful for brain science and neuron science such as functional cognition, brain wave-based control and human-computer interaction, etc. The combination of computer science and neuroscience promote scientists to solve more and more problems about human life such as how to use human brain (i.e. consciousness) to control their behavior, to control machines or even people^24^. Even more amazing is that the brain wave control could be from a cloud computing service, which makes the Internet be the extension of people’s brain. Our cell phone based wireless ECoG system has clearly demonstrated the capability of recording 32-channel neuron signals, and interacting with cloud system through APP of a cell phone, and performing electrical stimulation to treat epilepsy. The ECoG system has the capability for wireless communication, powerful signal processing and pattern recognition, yet the device is very small and portable, thus it has great potential for the above mentioned applications.

In summary, we have developed a new wireless brain-cell phone interaction ECoG system to record electrical signal of brain activities. The flexible ECoG microelectrode array consists of 32 channels which can fit conformally on wrinkle structure of cortex with high spatial resolution. Corresponding electronic circuits have also been developed for signal acquisition, processing and wireless communication with cell phone. The *in-vivo* experiments on a rate have clearly shown the flexible ECoG system can record, process and transmit electrical signals of brain activities to cell phone with good SNR and signal integrity, and demonstrated epilepsy by electrical stimulation through the ECoG microelectrodes. Compared with others, our system has many advantages, including higher sampling rate, larger bandwidth, lower power consumption, high transmission speed and long communication distance, thus demonstrated its great potential for future applications.

## Methods

All procedures conformed to the Guide for the Care and Use of Laboratory Animals (China Ministry of Health) and had been approved by Zhejiang University Committee on animal usage. Sprague Dawley rats weighing between 250–350 g were used in our experiments.

### Fabrication of flexible microelectrode array

The electrode device has an array of 32 microelectrodes, with a total width of 16.3 mm and length of 24.8 mm made on a PI film. The fabrication process is as follows: A (Polymethyl methacrylate) (PMMA) layer was spun coated on a glass substrate as the sacrificial layer for removal from the glass which was used as a support for easy fabrication, and was baked at 180 °C for 30 min; then a 60 μm PI layer was coated on top and baked at 230 °C for 180 min. After baking, microelectrodes were formed by photolithography and lift-off process. A Cr layer of 20 nm thickness, Ag of 600 nm and Cr of 20 nm were deposited by sputtering in sequence. Both the bottom and top Cr layers were used to improve adhesion of metal with the PI layers. A 600 nm thickness of Ag layer was used to achieve low impedance for better signal acquisition with low noise, yet to have sufficient flexibility. Then, another PI layer about 10 μm was coated on top of the Cr layer for insulation and baked at 230 °C for 180 min. The microelectrode array were patterned and etched by oxygen plasma to open windows of metal layer for electrical contact and holes for drug injection. Au of 60 nm thickness was then electroplated on the windows and holes of the Cr top layer to achieve better contact with neurons and to improve the conductivity of the microelectrodes. Once completed, through acetone steeping, the ECoG electrode with the PI layer was removed from the glass substrate using the PMMA as the sacrificial layer. A flexible printed circuit (FPC) board connector was used to connect the 32 microelectrodes of the ECoG electrode device.

### Development of electronic circuits

Electronic circuits were developed for signal acquisition, processing and wireless communication with cell phone. Lower power consumption BLE was used for wireless communication and powered with a button-type battery. A TI CC2541 BLE chip was used as the microcontroller unit (MCU) to control the function of RHD2132 (Digital Electrophysiology interface chips). Details of the circuit chip can be found from Figure S3 in Supplement. Balun circuit was used to convert differential signals to single-ended signals and for impedance matching. The decoupling capacitors were applied to filter spikes in signals, and the shunt capacitors to filter high-frequeData and materials availabilityncy noises. A 32 MHz crystal oscillator was used for normal operation, while the 32 kHz crystal oscillator for sleep mode; An Invert-F antenna with a central frequency of 2.4 GHz and 50 Ω impedance was designed for wireless communication. The designed wireless transmission distance is 10 m for this work, and can be easily upgraded according to applications.

### Data and materials availability

All data needed to evaluate the conclusions in the paper are present in the paper and/or the Supplementary information. Additional data related to this paper may be requested from the authors^24^.

## Electronic supplementary material

Portable wireless electrocorticography system with a flexible microelectrodes array for epilepsy treatment
