# Supplementary material for: Portable wireless electrocorticography system with a flexible microelectrodes array for epilepsy treatment
Source: Sci Rep. 2017 Aug 10;7:7808. doi: 10.1038/s41598-017-07823-3 (PMC5552815; doi:10.1038/s41598-017-07823-3)
Supplement: Supplementary file 1 — Portable wireless electrocorticography system with a flexible microelectrodes array for epilepsy treatment [file 41598_2017_7823_MOESM1_ESM.docx]

**Supplementary information**

**Portable wireless electrocorticography system with a flexible microelectrodes array for epilepsy treatment**

Kejun Xie^1^, Shaomin Zhang^3, 4^, Shurong Dong^1^, Shijian Li^2*^, Chaonan Yu^3, 4^, Kedi Xu^3, 4^, Wanke Chen­^1^, Wei guo^1^, Jikui Luo^5,6^, Zhaohui Wu^2^

1. Key Laboratory of Micro-nano Electronic Devices and Smart SystemKey Laboratory of Micro-nano Electronic Devices and Smart Systems of Zhejiang Province, College of

Information Science & Electronic Engineering, Zhejiang University, Hangzhou 310027, China;

1. College of Computer Science, Zhejiang University, Hangzhou 310027, China;
2. Key Laboratory of Biomedical Engineering of Education Ministry, Department of Biomedical Engineering, Zhejiang University, Hangzhou 310027, China;
3. Qiushi Academy for Advanced Studies (QAAS), Zhejiang University, Hangzhou 310027, China;
4. College of Electron Infor., Hangzhou Dianzhi University, 2^nd^ Street, Hangzhou 310018, China.
5. Inst. of Renew. Energ. & Environ. Technol., University of Bolton, Deane Road, Bolton BL3 5AB, United Kingdom


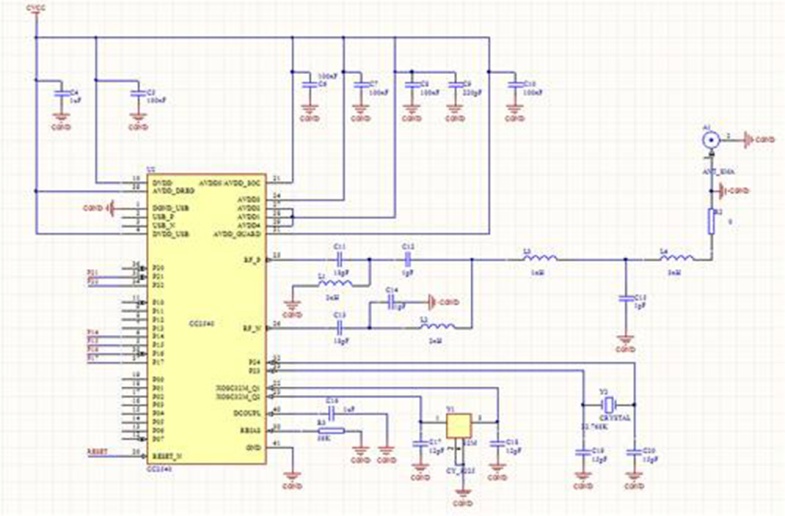


Figure S1. Schematic diagram of CC2541 as MCU; It needs Two crystal oscillators. 32MHz crystal oscillators works for normal operation mode while 32.768KHz works for sleep mode. CC2541 outputs differential signal. Inverted-F antenna is single-ended. So balun circuit is used to change differential signal to single-ended. Also balun circuit achieves impedance matching through changing the value of resistances and capacitances.


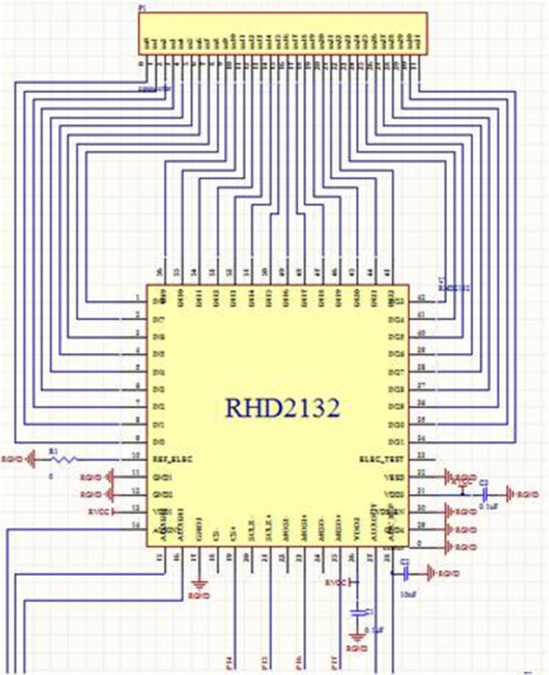


Figure S2. The RHD2132 pre-amplifier which has 32 channels of input, a 16-bit ADC, and an industry-standard SPI. The ADC has an operation of 1.05 M samples per second. Port elec_test was used to inject current for electrode stimulation


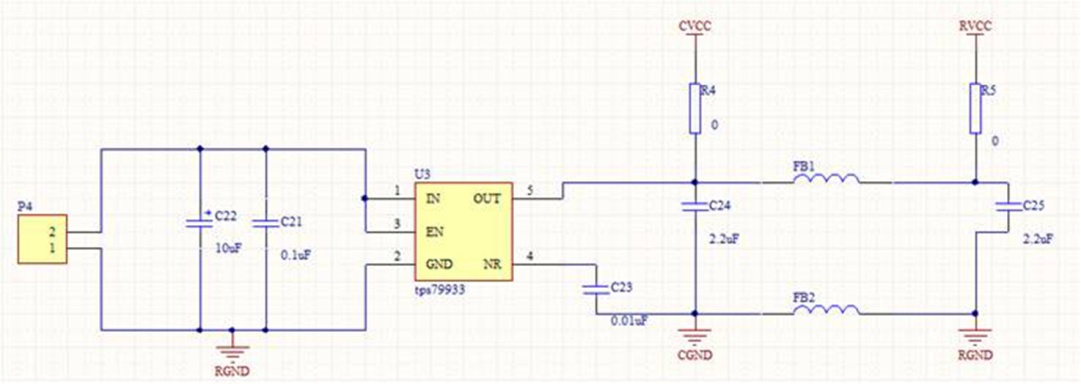


Figure S3. Schematic diagram of LDO circuit; This circuit can stabilize voltage at 3.3V. CVCC is used for CC2541 while RVCC for RHD2132. We use two 0Ω resistances to control if CVCC or RVCC are joined up with the circuit. So that we can test each module independently.


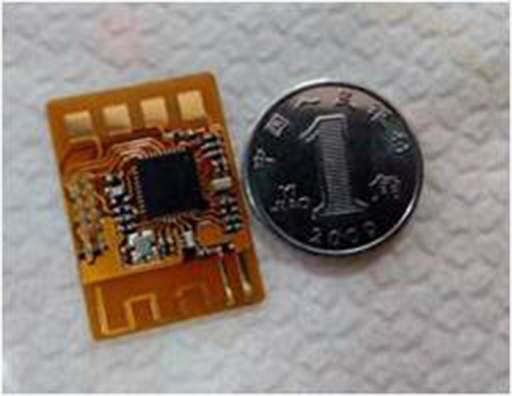


Figure S4. flexible printed circuit board; it’s made with polyimide(PI). CC2541 and RHD 2132 are on the different side of the PI board in order to save space. Compared to a coin, it has a small size.


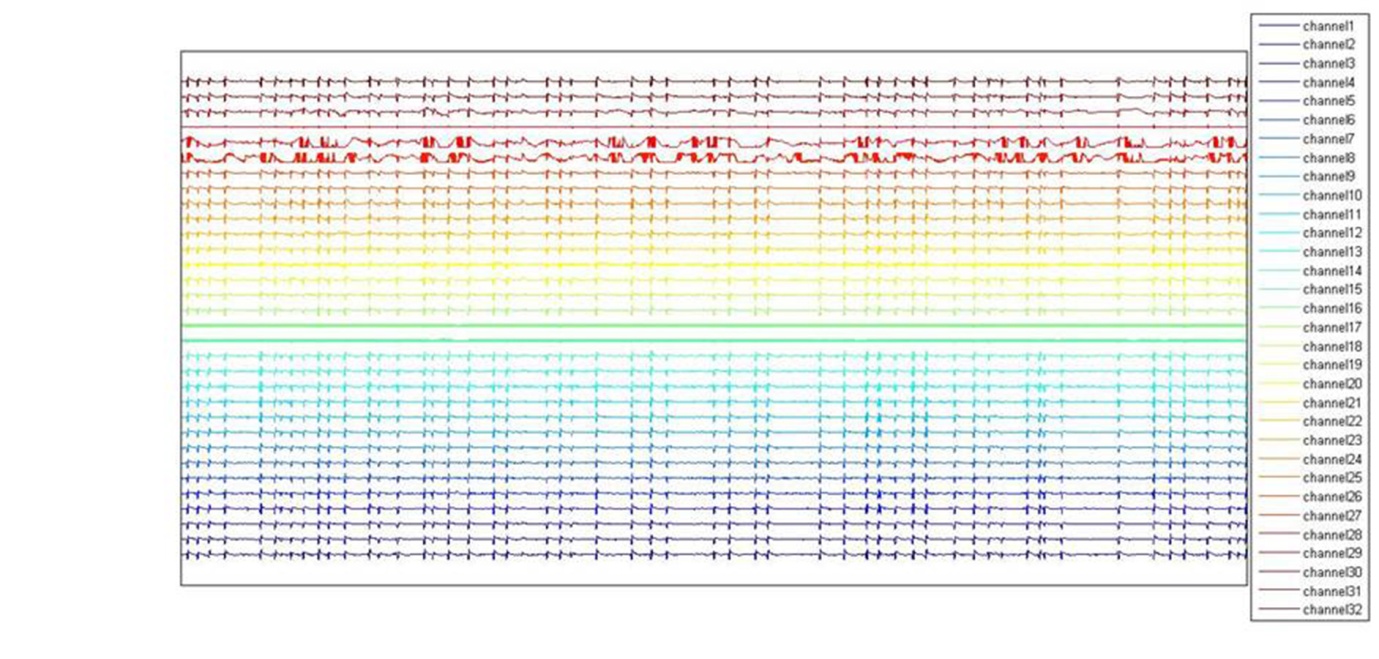


Figure S5. 32 channels of ECoG signal from a rat’s brain. We can see clearly that channel5 and channel6 have strongest signals. It’s supposed that this area is the nidus of epilepsy which is most active.


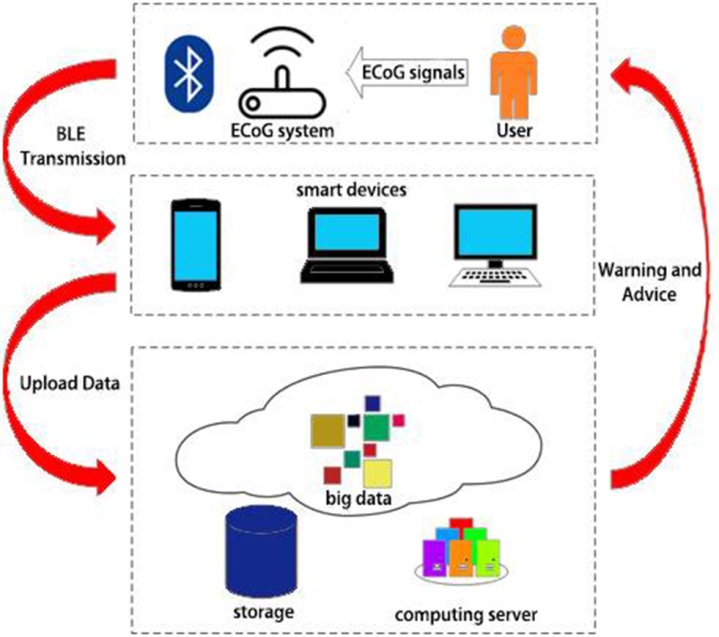


Figure S6. cloud model to process and recognize the real meaning of brain signals, then make an action based on proposed wireless ECoG system with flexible microelectrodes array
